# A Meta-Analysis of the Effects of Foam Rolling on Performance and Recovery

**DOI:** 10.3389/fphys.2019.00376

**Published:** 2019-04-09

**Authors:** Thimo Wiewelhove, Alexander Döweling, Christoph Schneider, Laura Hottenrott, Tim Meyer, Michael Kellmann, Mark Pfeiffer, Alexander Ferrauti

**Affiliations:** ^1^Faculty of Sport Science, Ruhr University, Bochum, Germany; ^2^Institute of Sports and Preventive Medicine, Saarland University, Saarbrücken, Germany; ^3^School of Human Movement Studies and School of Psychology, The University of Queensland, Brisbane, QLD, Australia; ^4^Department for Theory and Practical Performance in Sports, Institute of Sports Science, Johannes-Gutenberg University, Mainz, Germany

**Keywords:** rolling massage, sprint, jump, strength, flexibility, muscle pain

## Abstract

Foam rolling is thought to improve muscular performance and flexibility as well as to alleviate muscle fatigue and soreness. For this reason, foam rolling has become a popular intervention in all kinds of sport settings used to increase the efficiency of training or competition preparation as well as to speed post-exercise recovery. The objective of this meta-analysis was to compare the effects of foam rolling applied *before* (pre-rolling as a warm-up activity) and *after* (post-rolling as a recovery strategy) exercise on sprint, jump, and strength performance as well as on flexibility and muscle pain outcomes and to identify whether self-massage with a foam roller or a roller massager is more effective. A comprehensive and structured literature search was performed using the PubMed, Google Scholar, PEDro, and Cochrane Library search engines. Twenty-one studies were located that met the inclusion criteria. Fourteen studies used pre-rolling, while seven studies used post-rolling. Pre-rolling resulted in a small improvement in sprint performance (+0.7%, *g* = 0.28) and flexibility (+4.0%, *g* = 0.34), whereas the effect on jump (−1.9%, *g* = 0.09) and strength performance (+1.8%, *g* = 0.12) was negligible. Post-rolling slightly attenuated exercise-induced decreases in sprint (+3.1%, *g* = 0.34) and strength performance (+3.9 %, *g* = 0.21). It also reduced muscle pain perception (+6.0%, *g* = 0.47), whereas its effect on jump performance (−0.2%, *g* = 0.06) was trivial. Of the twenty-one studies, fourteen used foam rollers, while the other seven used roller massage bars/sticks. A tendency was found for foam rollers to offer larger effects on the recovery of strength performance (+5.6%, *g* = 0.27 vs. −0.1%, *g* = −0.01) than roller massagers. The differences in the effects between foam rolling devices in terms of pre-rolling did not seem to be of practical relevance (overall performance: +2.7 %, *g* = 0.11 vs. +0.4%, *g* = 0.21; flexibility: +5.0%, *g* = 0.32 vs. +1.6%, *g* = 0.39). Overall, it was determined that the effects of foam rolling on performance and recovery are rather minor and partly negligible, but can be relevant in some cases (e.g., to increase sprint performance and flexibility or to reduce muscle pain sensation). Evidence seems to justify the widespread use of foam rolling as a warm-up activity rather than a recovery tool.

## Introduction

In recent years, foam rolling has become a common practice in all kinds of sport settings and is highly regarded within the strength and conditioning field for increasing the efficiency of training or competition preparation and for accelerating post-exercise recovery (Healey et al., 2014; Jones et al., 2015; Monteiro and Neto, 2016). Foam rolling (FR) is a form of self-massage in which the targeted musculature is rolled and compressed utilizing a FR device (Peacock et al., 2014). Common FR tools include the foam roller and various types of roller massage bars/sticks, which come in several sizes and foam densities.

With foam rollers, athletes use their bodyweight to apply pressure to the soft tissues during the rolling motion, while roller massagers are applied with the upper extremities to the target muscles (Cheatham et al., 2015). The motions place both direct and sweeping pressure on the soft tissue, stretching it and generating friction between it and the FR device. Consequently, FR can be considered a form of self-induced massage because the pressure that the roller exerts on the muscles resembles the pressure exerted on the muscles through manual manipulation by the user himself (Pearcey et al., 2015). Some reasons why self-massage through FR has become a popular intervention technique used by both elite athletes and recreationally active individuals may be its affordable, easy, and time-efficient applicability as well as its close relationship to massage, which in turn is believed to benefit athletes by enhancing performance and recovery (Weerapong et al., 2005).

However, despite the popularity of FR, no consensus exists on its benefits (Cheatham et al., 2015; Pearcey et al., 2015). This may be partly due to the fact that few studies have examined the underlying physiological mechanisms of FR. Nevertheless, the potential effects of FR have been attributed to mechanical, neurological, physiological, and psychophysiological parameters (Aboodarda et al., 2015; Cavanaugh et al., 2017; Monteiro et al., 2018; Phillips et al., 2018). The mechanical mechanisms are comprised of a number of sub-mechanisms, such as reduction in tissue adhesion, altered tissue stiffness, and thixotropic responses (Aboodarda et al., 2015; Kelly and Beardsley, 2016). Within neurological models, it is theorized that FR may potentiate analgesic effects and muscular recovery by mediating pain-modulatory systems (e.g., nociceptor and mechanoreceptor sensitivity and/or diffuse noxious inhibitory control) (Cavanaugh et al., 2017; Jo et al., 2018). The proposed physiological mechanisms are increased blood flow and parasympathetic circulation, as well as inflammatory responses and associated trigger-point break down (Aboodarda et al., 2015; Kelly and Beardsley, 2016). Psychophysiological responses may include improved perceptions of well-being and recovery due to the increase of plasma endorphins, decreased arousal level, an activation of the parasympathetic response and/or placebo effect (Weerapong et al., 2005; Phillips et al., 2018).

Due to the potential underlying physiological mechanisms, it is believed that FR can improve both acute athletic performance as well as recovery from an intensive bout of physical activity (Cheatham et al., 2015). Therefore, studies on the effects of FR have either determined whether massage-like mechanical pressure with a foam roller or roller massager *prior* to activity affects muscle performance (i.e., pre-rolling as a warm-up activity), or whether FR *after* an intense bout of exercise enhances muscle recovery (i.e., post-rolling as a recovery tool). Unfortunately, the literature on FR that does exist is equivocal and insufficient, which is why the widespread use of FR is to date not fully supported by the available empirical data. In addition, there is currently no meta-analysis that has evaluated the literature and calculated the pooled effects of FR. This creates a gap in the translation from research to practice for strength and conditioning coaches who use FR tools and recommend these products to their athletes (Cheatham et al., 2015). Accordingly, the aims of the study were to conduct a meta-analytical review of the effects of pre-rolling and post-rolling on performance, flexibility, and muscle pain outcomes in healthy and physically active individuals and to identify whether self-massage with a foam roller or a roller massager is more effective.

## Methods

### Search Strategy

**Figure 1:**
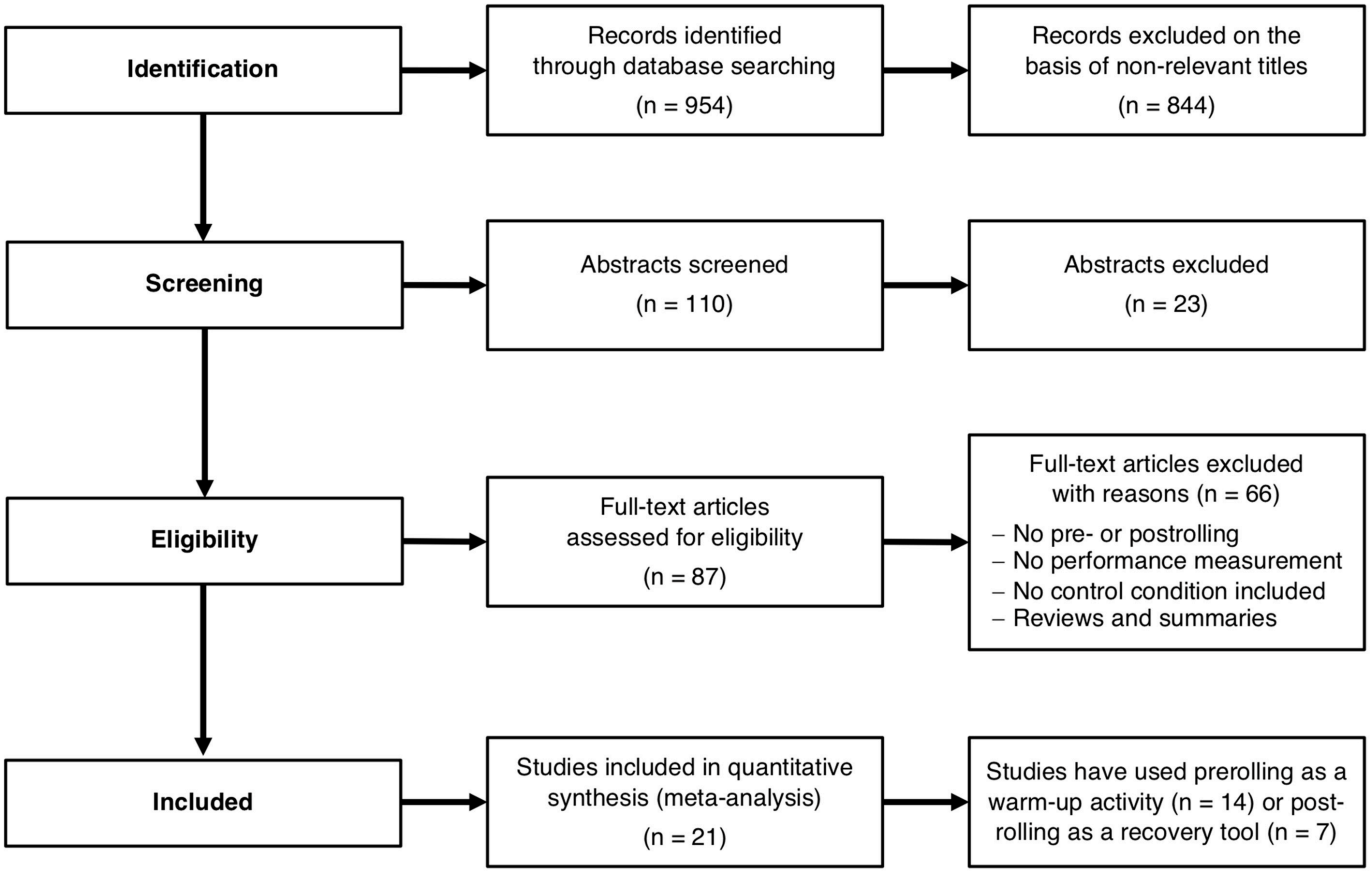
Overview of the selection process for the studies included in this meta-analysis. *N* indicates the number of studies.

A comprehensive and structured search of articles were performed using the PubMed, Google Scholar, PEDro, and Cochrane Library search engines. Different sets of seven key terms (“self-massage,” “foam rolling,” “roller massage,” “roller massager,” “self-myofascial release,” “performance,” “recovery”) were combined by Boolean logic (“AND,” “OR”), and the results were limited to human subjects that were healthy and physically active as well as to articles written in English. Each database was searched from the earliest available article up to December 2017. We also searched the reference lists of all incoming articles and extracted the appropriate publications. From the 954 abstracts reviewed, 110 potentially suitable articles were identified (Figure 1).

### Selection Criteria

The selection of articles for inclusion in this meta-analysis was based on the following criteria. First, only publications that appeared in an international, peer-reviewed scientific journal were selected. Second, a FR intervention had to have been done as part of the analysis, regardless of which type of FR device was used for the intervention. Third, the FR intervention had to have been used either as a warm-up or a recovery routine. Fourth, before and after the FR intervention, measurements of performance, flexibility, and/or muscle pain outcomes had to have been conducted. Fifth, there had to have been a control condition, where athletes were subdivided either as their own controls or randomly into an intervention and control group. The first author was responsible for the study selection. After the selection process, all studies were discussed among three authors. In case of disagreement about the inclusion of a study, a voting process was used to determine if a study should be included or not. Figure 1 provides a flow chart of the literature search.

### Classification and Quality Assessment of the Studies

The inclusion criteria were met by 21 studies, 14 of which used pre-rolling as an exercise warm-up routine, while seven used post-rolling to enhance recovery mechanisms. For further analysis, the studies were categorized according to the type of FR device used (i.e., foam roller or roller massager). Several studies were included more than once in the analysis. This was the case, for example, when several follow-up examinations were carried out (e.g., after 24 and 48 h) or several types of performance indicators were measured. The Cochrane risk of bias tool (Higgins et al., 2011) was used to assess the quality of each included study.

### Statistical Analysis and Assessment of Effect Sizes

A standardized form was used to extract all relevant data and important methodological details from the studies. For each study, relative changes in performance, flexibility, and muscle pain were calculated for the treatment condition and the control condition. By subtracting the two values, the net effect of the treatment on changes in performance, flexibility, and muscle pain was calculated. Effect sizes (ES, Hedges' *g* values) were estimated according to the following formula:

$$g=c_{P}\;\frac{\left(M_{post,\;foam\;rolling}-\;M_{pre,\;foam\;rolling}\right)\;\left(M_{post,\;control}-\;M_{pre,\;control}\right)}{SD_{pre}}$$

where *c*_*p*_ is a bias factor recommended for small sample sizes (Morris, 2008), *M*_*pre, foamrolling*_*, M*_*post, foamrolling*_*, M*_*pre, control*_, and *M*_*post, control*_ are the respective mean values of performance, flexibility, and muscle pain, and *SD*_*pre*_ is the pooled pre-test standard deviation. This method was chosen because it has been suggested for the ES calculation of controlled pre-test-post-test study designs in meta-analyses (Higgins et al., 2011). Negative effects on performance, flexibility, and muscle pain are marked with a minus sign ES deviations and 95% confidence intervals were calculated as described by Borenstein et al. (2011). In addition, the ES was converted to percentiles as described by Coe (2002). For example, an ES of 0.5 means that the score of the average subject in the FR group is 0.5 standard deviations above the average subject in the control group, and hence exceeds the score of 69%. The value of 69% indicates that the average subject in the FR group would score higher than 69% of the control group that was initially equivalent.

If more than one parameter of performance was measured, a combined effect was calculated by averaging the relative change and the ES, and calculating the combined ES variance (Borenstein et al., 2011). In this context, a correlation coefficient of 0.9 was used based on the values reported in studies by Harbo et al. (2012) and Nuzzo et al. (2008).

The total results for the analyzed conditions were determined by the calculation of inverse-variance-weighted *g*-values (Borenstein et al., 2011). For nine of the twenty-one studies, more than one result was included in the analysis because several follow-up examinations were performed (e.g., after 24 and 48 h). In these cases, the respective results were combined as described above, assuming correlation coefficients of 0.9. To combine the different types of sprint, jump, and strength performances, a correlation coefficient of 0.6 was used.

The data of each individual study as well as weighted-average values are presented in forest plots. The magnitude of *g* was categorized according to Cohen (1992) (i.e., 0.00–0.19 = negligible effect, 0.20–0.49 = small effect, 0.50–0.79 = moderate effect, ≥0.80 = large effect). The values are given with 95% confidence intervals to express the uncertainty of the true effect. ES can be interpreted as evidence of the benefit of pre-rolling or post-rolling when the average and 95% confidence intervals are above zero.

## Results

### Included Studies

**Figure 2:**
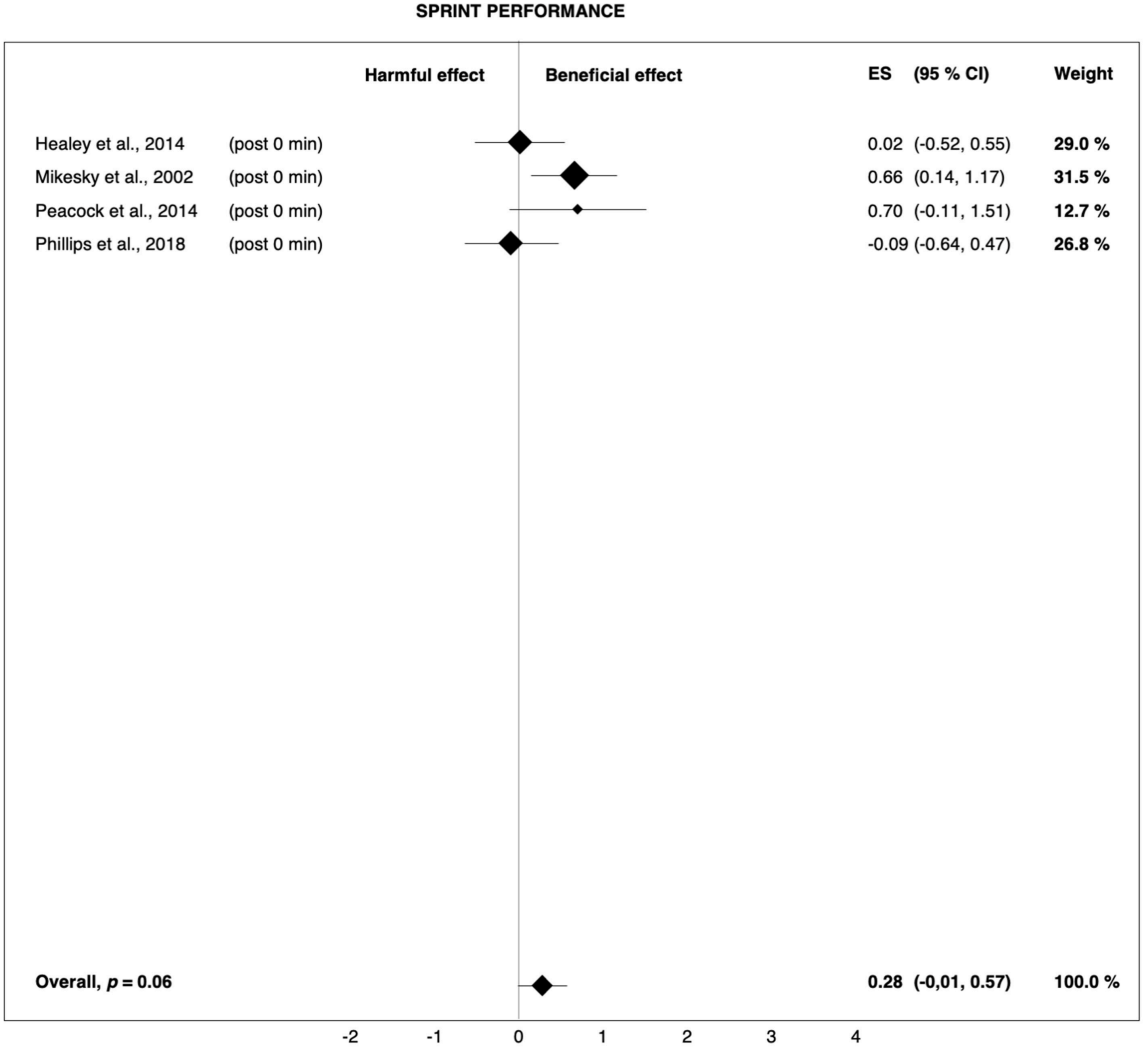
Forest plot summarizing the effects of pre-rolling on sprint performance. For each study, the timing of the post-test is included in parentheses. The studies are sorted by increasing the duration between the foam rolling intervention and the post-test. The rectangles represent the weighted effect size (ES) and the lines are the 95% confidence intervals (CI). The size of the rectangles indicates the weight of the study.

**Figure 3:**
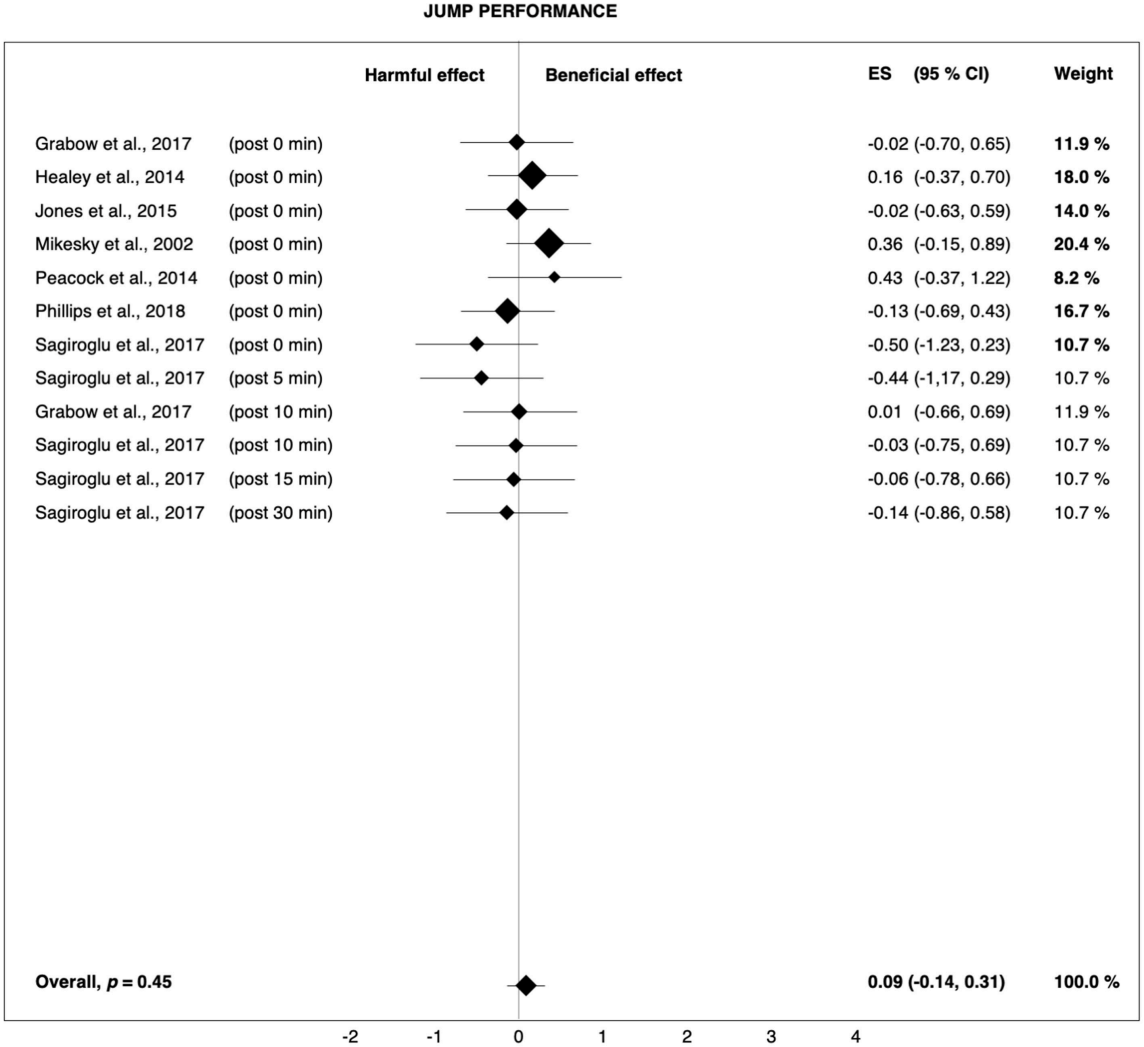
Forest plot summarizing the effects of pre-rolling on jump performance. For each study, the timing of the post-test is included in parentheses. The studies are sorted by increasing duration between the foam rolling intervention and the post-test. The rectangles represent the weighted effect size (ES) and the lines are the 95% confidence intervals (CI). The size of the rectangles indicates the weight of the study.

**Figure 4:**
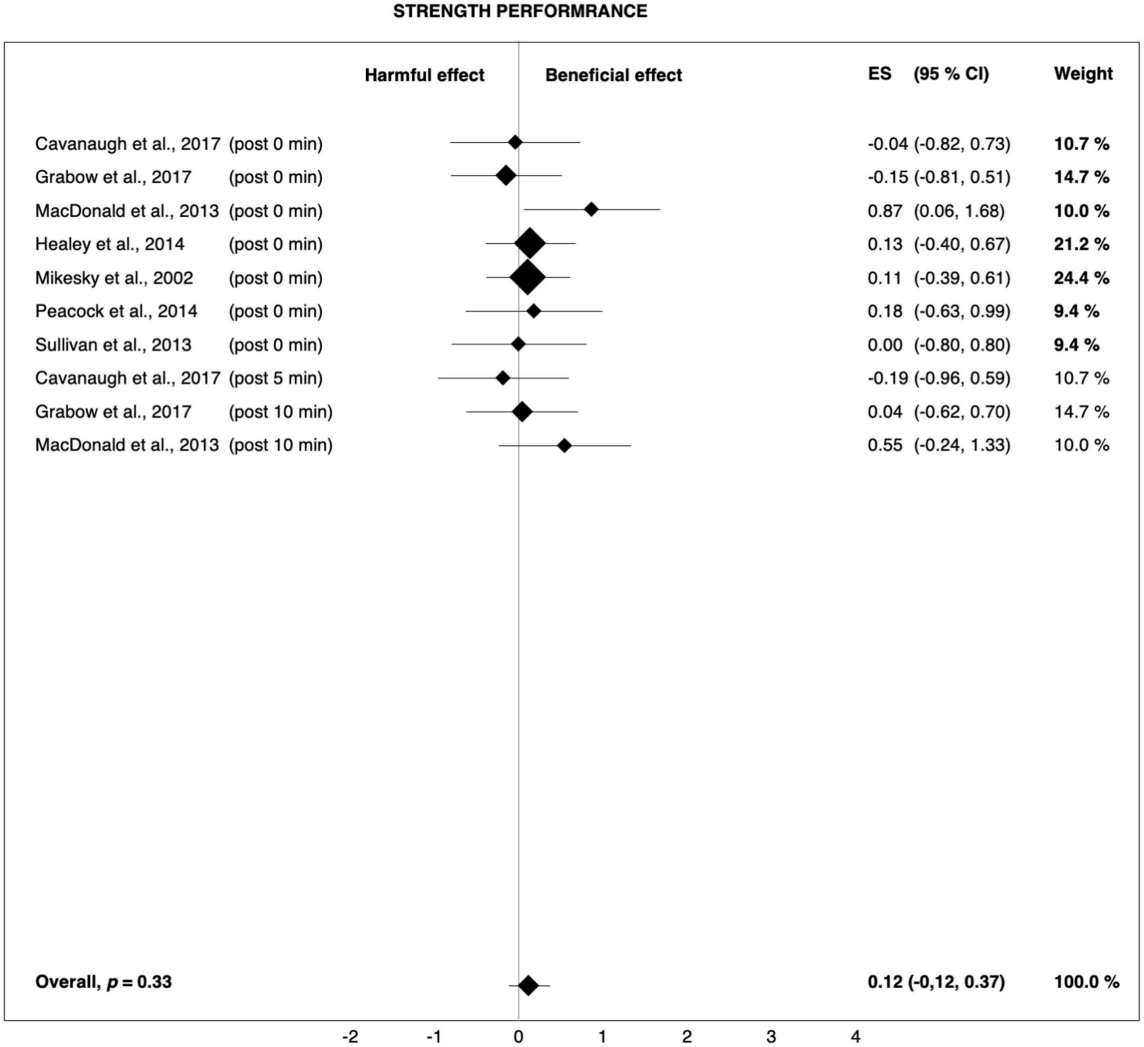
Forest plot summarizing the effects of pre-rolling on strength performance. For each study, the timing of the post-test is included in parentheses. The studies are sorted by increasing the duration between the foam rolling intervention and the post-test. The rectangles represent the weighted effect size (ES) and the lines are the 95% confidence intervals (CI). The size of the rectangles indicates the weight of the study.

**Figure 5:**
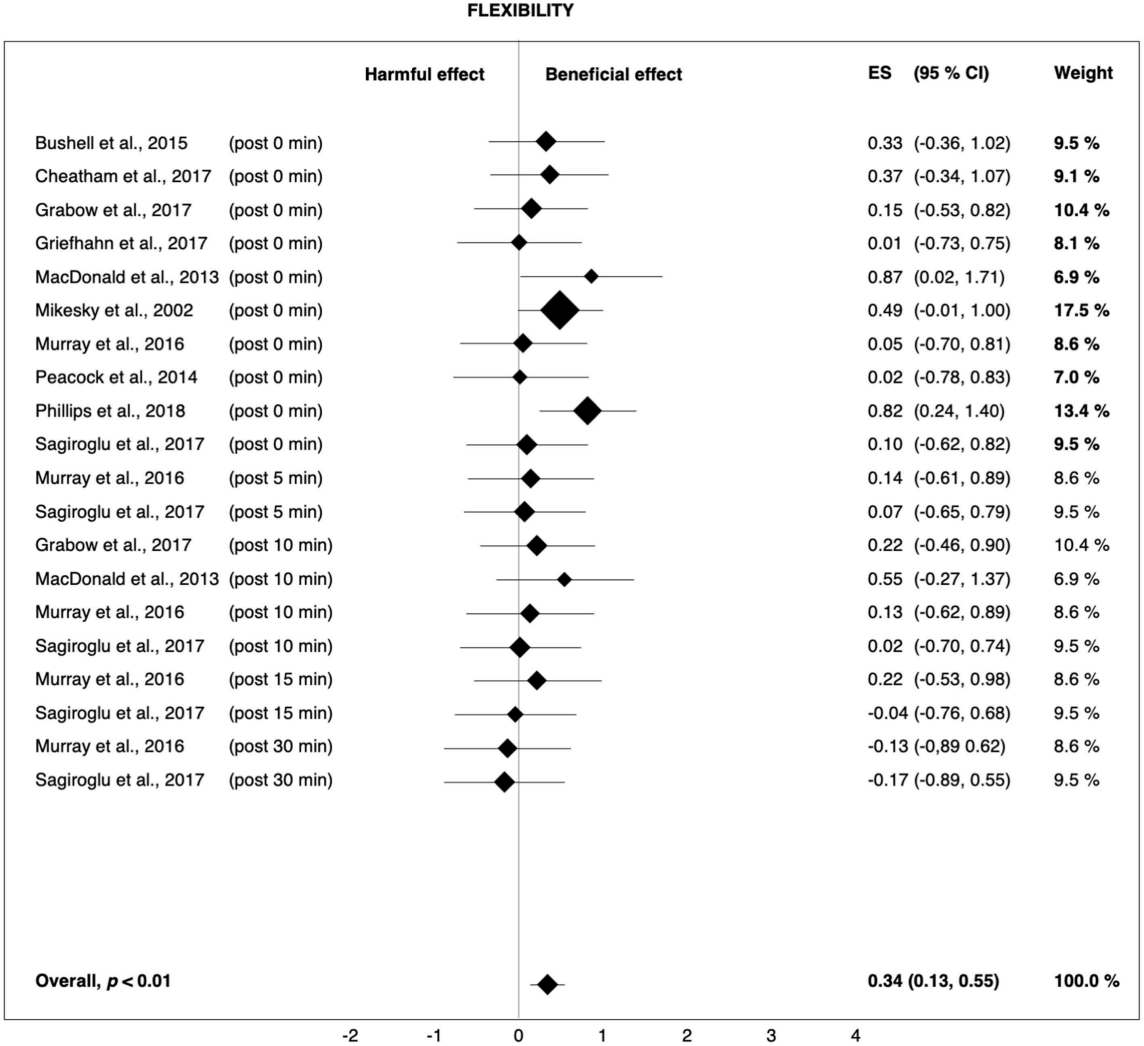
Forest plot summarizing the effects of pre-rolling on flexibility. For each study, the timing of the post-test is included in parentheses. The studies are sorted by increasing the duration between the foam rolling intervention and the post-test. The rectangles represent the weighted effect size (ES) and the lines are the 95% confidence intervals (CI). The size of the rectangles indicates the weight of the study.

**Figure 6:**
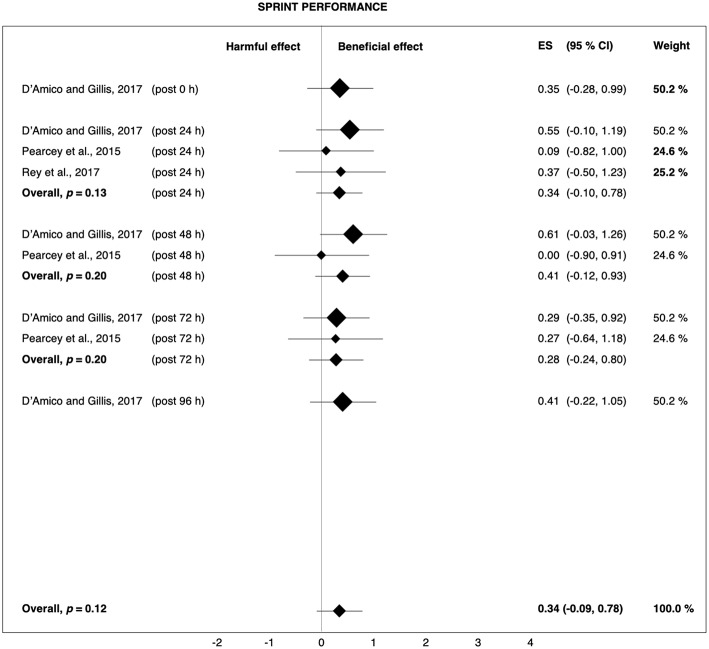
Forest plot summarizing the effects of post-rolling on sprint performance. For each study, the timing of the post-test is included in parentheses. The studies are sorted by increasing the duration between the foam rolling intervention and the post-test. The rectangles represent the weighted effect size (ES) and the lines are the 95% confidence intervals (CI). The size of the rectangles indicates the weight of the study.

**Figure 7:**
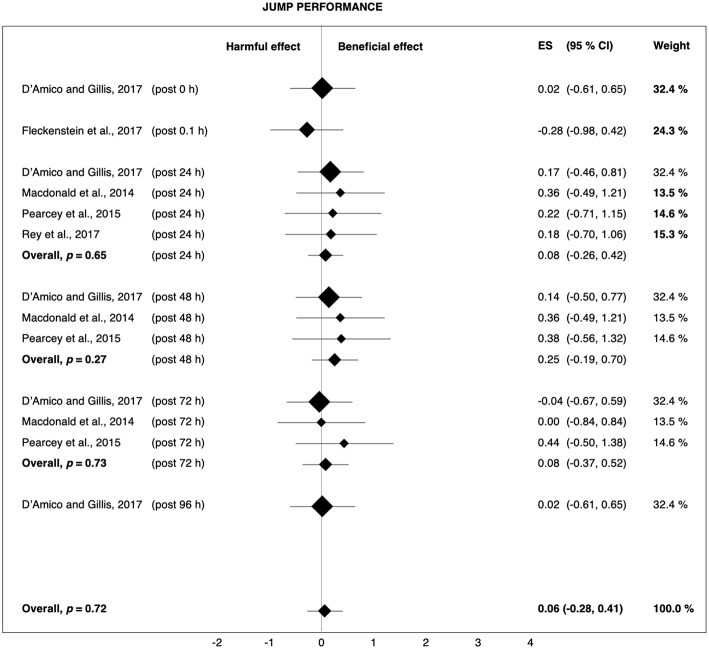
Forest plot summarizing the effects of post-rolling on jump performance. For each study, the timing of the post-test is included in parentheses. The studies are sorted by increasing the duration between the foam rolling intervention and the post-test. The rectangles represent the weighted effect size (ES) and the lines are the 95% confidence intervals (CI). The size of the rectangles indicates the weight of the study.

**Figure 8:**
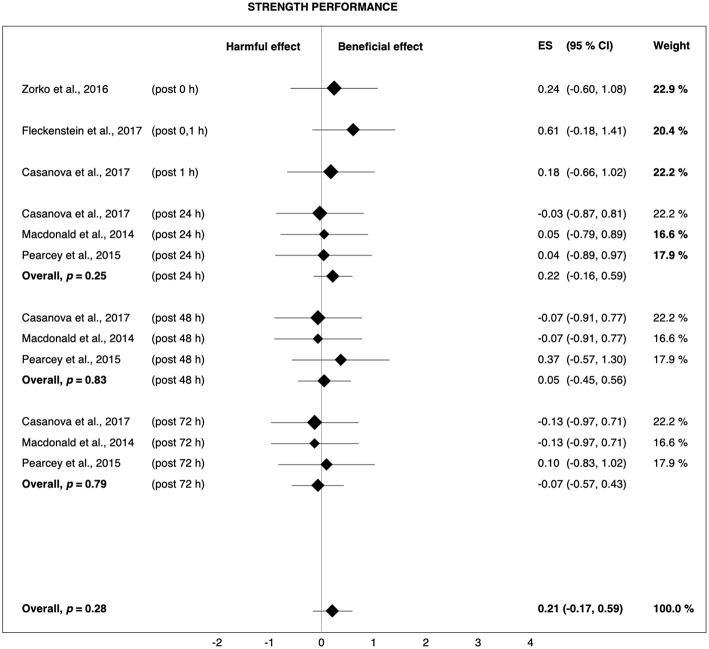
Forest plot summarizing the effects of post-rolling on strength performance. For each study, the timing of the post-test is included in parentheses. The studies are sorted by increasing the duration between the foam rolling intervention and the post-test. The rectangles represent the weighted effect size (ES) and the lines are the 95% confidence intervals (CI). The size of the rectangles indicates the weight of the study.

**Figure 9:**
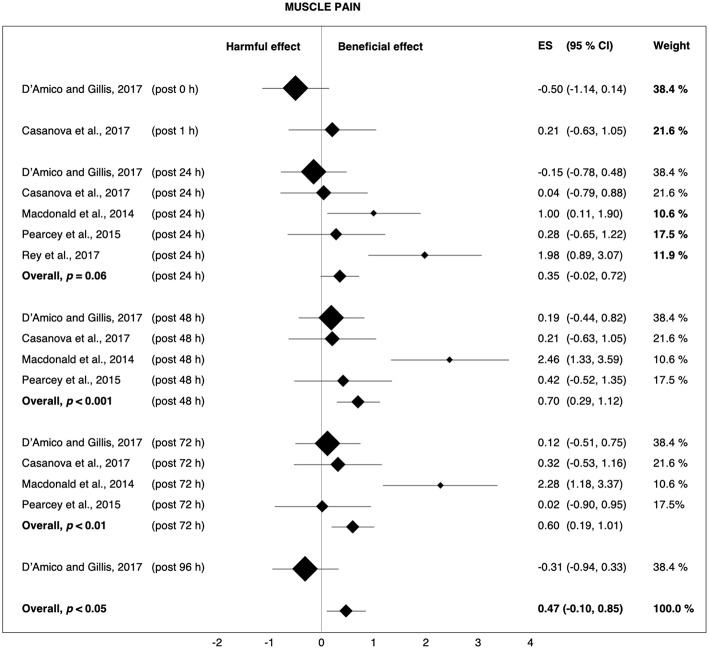
Forest plot summarizing the effects of post-rolling on muscle pain. For each study, the timing of the post-test is included in parentheses. The studies are sorted by increasing the duration between the foam rolling intervention and the post-test. The rectangles represent the weighted effect size (ES) and the lines are the 95% confidence intervals (CI). The size of the rectangles indicates the weight of the study.

Twenty-one studies with a total number of 454 subjects met the inclusion criteria, fourteen of which used pre-rolling as an exercise warm-up strategy (*n* = 306), while seven used post-rolling to enhance recovery (*n* = 148). Of the twenty-one studies, fourteen used foam rollers, while the other seven used roller massage bars/sticks. The characteristics of the included studies are summarized in Supplementary Table 1 (studies using pre-rolling) and Supplementary Table 2 (studies using post-rolling). The calculated ES for the effects of FR on performance, flexibility, and muscle pain outcomes are shown in Figures 2–**9**.

The use of the Cochrane risk of bias tool (Higgins et al., 2011) showed a comparable bias level for most of the included studies. Regarding selection bias, almost all studies mentioned a random assignment of their subjects into either a FR or control group. Accordingly, the risk of selection bias was considered low. However, in the research article by Sullivan et al. (2013), it was not explicitly stated how the participants were assigned to the different groups. Here, the selection bias remained unclear. The blinding of the subjects was not possible due to the nature of the FR technique. Consequently, the risk of a placebo bias was comparatively high. Mikesky et al. (2002) imposed blinding on researchers and participants during testing. Griefahn et al. (2017) stated that only the examiners were blinded during outcome assessments, whereas Cheatham et al. (2017) imposed blinding only on subjects. None of the other research articles provided any information on blinding. Regarding attrition bias, only one study reported on drop-outs (*n* = 2; Bushell et al., 2015).

Furthermore, to minimize possible learning effects, twelve studies (Mikesky et al., 2002; MacDonald et al., 2013; Healey et al., 2014; Jones et al., 2015; Pearcey et al., 2015; Zorko et al., 2016; Cavanaugh et al., 2017; Cheatham et al., 2017; D'Amico and Gillis, 2017; Grabow et al., 2017; Casanova et al., 2018; Phillips et al., 2018) provided participants an organized familiarization with performance tests prior to the first testing session; in nine studies (MacDonald et al., 2013; Peacock et al., 2014; Jones et al., 2015; Pearcey et al., 2015; Cheatham et al., 2017; D'Amico and Gillis, 2017; Grabow et al., 2017; Casanova et al., 2018; Phillips et al., 2018), the participants were instructed to avoid strenuous exercise before and/or during the experimental period; in seven studies (MacDonald et al., 2013; Pearcey et al., 2015; Cavanaugh et al., 2017; D'Amico and Gillis, 2017; Grabow et al., 2017; Rey et al., 2017; Phillips et al., 2018), diet control was mentioned and/or the subjects were asked to maintain their normal dietary intake and to refrain from nutritional supplements and alcohol intake during the experimental period; and in six studies (Mikesky et al., 2002; Macdonald et al., 2014; Zorko et al., 2016; Cavanaugh et al., 2017; Cheatham et al., 2017; Rey et al., 2017), it was explicitly stated that each participant was always examined at approximately the same time of day.

Overall, based on the quality assessment of the studies included in this meta-analysis, none of the studies was considered to have a high risk of bias, except for the high risk of placebo bias that can be inevitable for this kind of studies.

### Pre-rolling

Pre-rolling resulted in a small improvement in sprint performance (+0.7%, *g* = 0.28) and flexibility (+4.0%, *g* = 0.34), whereas the effect on jump (−1.9%, *g* = 0.09) and strength performance (+1.8%, *g* = 0.12) was negligible. The weighted-average overall performance change due to pre-rolling was +1.5% (*g* = 0.20). Of the fourteen studies investigating the effects of pre-rolling on performance and flexibility, 10 (MacDonald et al., 2013; Healey et al., 2014; Peacock et al., 2014; Bushell et al., 2015; Jones et al., 2015; Murray et al., 2016; Cheatham et al., 2017; Griefahn et al., 2017; Sagiroglu et al., 2017; Phillips et al., 2018) used a cylindrical foam roller (overall performance: +2.7%, *g* = 0.11; flexibility: +5.0%, *g* = 0.32), while the remaining four studies (Mikesky et al., 2002; Sullivan et al., 2013; Cavanaugh et al., 2017; Grabow et al., 2017) used a type of roller massage bar/stick (overall performance: +0.4%, *g* = 0.21; flexibility: +1.6%, *g* = 0.39).

### Post-rolling

Post-rolling slightly attenuated exercise-induced decreases in sprint (+3.1%, *g* = 0.34) and strength performance (+3.9%, *g* = 0.21). It also reduced muscle pain perception (+6.0%, *g* = 0.47), whereas the effect on jump performance (−0.2%, *g* = 0.06) was trivial. The weighted-average overall performance change in response to post-rolling was +2.0% (*g* = 0.19). The effects of post-rolling using a cylindrical foam roller (strength performance: +5.6%, *g* = 0.27; muscle pain: +6.0%, *g* = 0.55) were examined by four studies (Macdonald et al., 2014; Pearcey et al., 2015; Zorko et al., 2016; Fleckenstein et al., 2017), while the remaining three studies (D'Amico and Gillis, 2017; Rey et al., 2017; Casanova et al., 2018) used a type of roller massage bar/stick (strength performance: −0.1%, *g* = −0.01; muscle pain: +5.8%, *g* = 0.20).

## Discussion

There is a growing body of literature examining the use of FR as a warm-up activity (i.e., pre-rolling) or as a recovery strategy (i.e., post-rolling); however, the effectiveness of FR is still in question in both scenarios. The variation in methodological design, combined with the differences in FR intervention, exercise modality, and training status of the populations investigated, has perhaps contributed to the apparently inconsistent findings. This study used a meta-analytical approach to (1). Explore whether the use of pre-rolling and post-rolling are effective tools to improve sprint, jump, and strength performance as well as flexibility and muscle pain outcomes and (2). To identify whether self-massage with a foam roller or a roller massager is more effective. The results indicate that pre-rolling causes a small acute improvement in sprint performance and flexibility, while its effect on jump and strength performance was negligible. Second, when foam rolling is used as a recovery tool, participants experience slightly reduced decrements in sprint and strength performance and a small reduction in the severity of muscle pain. Third, a tendency was found for foam rollers to offer larger recovery effects than roller massagers, while the differences in the effects between FR devices in terms of pre-rolling did not seem to be of practical relevance.

### Pre-rolling

Relevant effect sizes for average improvements in performance due to pre-rolling were found only for sprinting. The total Hedges' *g* of 0.28 (Figure 2) indicates that with the use of pre-rolling, 58% of the population is likely to experience increased sprint performance (Coe, 2002). However, the average percentage improvement in sprint performance was only 0.7%. In this context, Hopkins et al. (1999) defined the smallest worthwhile performance enhancement (i.e., in the case of the present study, the minimum improvement making pre-rolling worthwhile) as the value increasing the chance of victory for an athlete by 10%. Based on this definition, they concluded that an enhancement as small as 0.3–0.4 of the within-athlete standard deviation known as the coefficient of variation (CV) is important for at least the best athletes. For sprinting, Malcata and Hopkins (2014) as well as Tanner and Gore (2013) reported CVs of ~0.8%. The smallest important change in sprint performance thus corresponds to ~0.3%. This shows that although the effect size was rather small from a purely statistical point of view, when within-athlete variability is taken into account, the average improvement in sprint performance induced by pre-rolling is within a range that is relevant for elite athletes.

However, for recreational athletes, a change in sprint performance as small as ~0.3% may be barely noticeable due to a likely greater within-athlete variability. In this case, the minimum worthwhile enhancement in sprint performance would be greater than the change in sprint performance induced by pre-rolling. Consequently, the effects of pre-rolling on sprint performance seem to be more relevant for elite athletes, while it is possible that recreationally active individuals may not benefit substantially from pre-rolling. Furthermore, the small average overall effect size for sprint performance is based on only four studies and is mainly due to the studies conducted by Mikesky et al. (2002) and Peacock et al. (2014) showing effect sizes of 0.66 and 0.70, respectively. Therefore, it can be speculated that sprint performance does not, per se, benefit more from pre-rolling than the other performance components and that the slightly larger effect sizes found for sprinting are, rather, due to methodological aspects and/or aberrations in the data. Consequently, sprint performance results should be interpreted with caution, as the number of available studies was limited and only two of them showed a clear positive effect.

Several potential physiological effects of FR could explain the trend of improved sprint performance following pre-rolling. One possibility is that FR immediately prior to sprinting breaks up what are known as barrier trigger-points (Bonci and Oswald, 1993). These are identified as inflexible bands of muscle containing knots resulting from muscle spasm. Barrier trigger-points are typically painless and can result in muscle weakness, muscle fatigue, and muscle stiffness (Mikesky et al., 2002). All of these factors could obviously have an impact on sprinting. FR may break up these trigger-points. Decreasing muscle spasms would not only decrease the amount of internal resistance to muscle movement, but also enable the previously spasmodic tissue to contribute to the athletic activity being performed (Mikesky et al., 2002). However, this explanation remains highly speculative, and there is no concrete evidence proving that the release of trigger-points makes FR effective.

Alternative explanations for acute benefits in performance could be a potential warm-up and/or placebo effect. Self-massage with a foam roller necessitates supporting one's partial body weight with the upper body, similar to with planking exercises. These exercises primarily involve isometrically holding the body in a prone position and are typically used to strengthen the core. Isometric exercises such as planking are in some ways similar to FR because the body position is maintained in an analogous manner, requiring similar isometric actions to support one‘s body weight. Planking in turn would have a warm-up effect through possible increased skin and muscle temperature, increased blood flow, and enhanced flexibility/mobility (Healey et al., 2014). Moreover, the observed effect of FR might have been confounded by a potential psychosomatic disorder, meaning the subjects may have performed better following FR treatment simply because they believed it would improve their performance (Jo et al., 2018).

It should also be noted that sprint performance mainly depends on muscle strength and neuromuscular coordination. However, similar to the findings of Cheatham et al. (2015), the effects of pre-rolling on jump (*g* = 0.09) and strength performance (*g* = 0.12) were negligible (Figures 3, 4). Thus, it cannot be definitively determined if the observed effects on sprint performance were really due to FR. For example, it is possible that the trend toward improved sprint performance was the result of a placebo effect, as FR can hardly be blinded. The reason the other measures of physical performance did not show trends toward improvements is unclear. Mikesky et al. (2002) suggested that the measures of jump capacity and strength are rather one-dimensional when compared with the complexity and coordination required to sprint. As such, large improvements in more isolated tasks are not as remarkable, while the combined effects on more complex, repetitive tasks become more evident. Although it is a coordinated task, jumping is so brief in duration, at least compared to sprinting, that any combined improvements are not afforded a chance to be revealed.

The largest average effect of pre-rolling was related to flexibility. The overall Hedges' *g* of 0.34 (Figure 5) indicates that 62% of the population will experience short-term improvements in flexibility when using pre-rolling as a pre-exercise warm-up (Coe, 2002). Cheatham et al. (2015) assumed that the effects of FR on flexibility would be attributed to the altered viscoelastic and thixotropic properties of the fascia (i.e., remobilizing the fascia back to a gel-like state), as well as increases in intramuscular temperature and blood flow due to the friction created by the foam roller and the mechanical breakdown of scar tissue. However, this is merely speculation by the authors and is not based on direct scientific observations. In addition, hypotheses related to the mechanisms of pressure-associated changes in myofascial properties have been questioned. The pressure that is required to deform firm fascial tissue is greater than the physical range that is usually achieved by FR (Schleip, 2003). Therefore, a change in the thixotropic property of the fascia surrounding the muscle may be more likely (Phillips et al., 2018). This change is possible because the fascia is composed of colloidal substances that can become more gelatinous when they encounter heat and mechanical stress (de Souza et al., 2019). However, in colloidal substances, the thixotropic effect only lasts as long as the pressure or heat is applied, and within minutes, the substance returns to its original gel state (Schleip, 2003). Therefore, it is unlikely that FR would have a sustained effect on flexibility by changing the thixotropic property of the fascia.

Apart from this, FR may increase flexibility due to a process known as autogenic inhibition. As the FR device applies pressure to the muscle tissue, it is believed that mechanoreceptors called Golgi tendon organs (GTO) send a message to the central nervous system that substantial tension is being placed on the muscle, causing the central nervous system to relax that muscle to prevent it from tearing (Larson, 2014). However, Edin and Vallbo (1990) found that GTOs were insensitive to the tension produced on the tendon through stretching. If stretch-induced GTO inhibition exists, it is more likely to occur with large-amplitude stretches and not from the small tensile forces that are exerted during FR. Furthermore, any possible GTO inhibition subsides almost immediately after the cessation of tension in the tendon (Behm, 2018). Therefore, it seems unlikely that this mechanism would contribute to increased flexibility following FR. The most plausible explanation for short-term improvements in flexibility could be the effect of FR on the central pain-modulatory systems. For example, constant and vigorous pressure exerted on the soft tissues may overload the skin receptors, thus inhibiting or minimizing pain sensation and increasing stretch tolerance (Kelly and Beardsley, 2016; de Souza et al., 2019). This hypothesis is supported by the findings of Aboodarda et al. (2015) and Cavanaugh et al. (2017) who have shown that FR can improve pain perception.

Apart from the study by Sagiroglu et al. (2017), which has shown that pre-rolling has a harmful effect on jump performance, the research suggests that pre-rolling may offer small short-term benefits in promoting flexibility without negatively affecting muscle performance. This is an important finding to consider when putting together a menu of warm-up activities, since training and competition preparation should always aim to enhance performance. Nevertheless, additional research is necessary to identify different FR protocols that are relevant to different sports and to develop guidelines that ensure FR routines do not impair performance. For example, despite causing an acute increase in range of motion in the joints, prolonged static stretching of more than 60 s per muscle is likely to result in significant performance impairment (Kay and Blazevich, 2012; Behm et al., 2015; Reid et al., 2018). Therefore, one might assume that prolonged static stretching is not recommended during pre-event warm-up activities, especially when performance is required immediately after stretching. However, in studies that conducted performance tests >10 min after static stretching, performance changes were typically statistically trivial unless extreme stretch protocols were used (Behm et al., 2015). Moreover, Blazevich et al. (2018) and Reid et al. (2018) reported that potential performance decrements caused by static stretching are insignificant with shorter stretching durations (i.e., <60 s) and appear to be resolved after a complete, progressive pre-exercise warm-up routine. Therefore, strong evidence supports the deleterious effects of static stretching prior to performance (Behm and Chaouachi, 2011), but when used properly, static stretching can promote flexibility and injury prevention without negatively affecting muscle performance (Behm et al., 2015; Reid et al., 2018).

### Post-rolling

The current review demonstrates that post-rolling recovers exercise-induced decreases in sprint and strength performance more quickly than passive recovery. The overall Hedges' *g* of 0.34 and 0.21 for sprint and strength (Figures 6, 8) indicate that 62 and 58%, respectively, of the population will experience the accelerated recovery of sprint and strength performance when using post-rolling (Coe, 2002). The average sprint and strength performance improvements were 3.1 and 3.9%, respectively, reflecting a range that is clearly higher than the smallest worthwhile change defined by Hopkins et al. (1999).

Prolonged impairments in muscular function have been attributed to a multifaceted process from central factors involving the central nervous system and nervous pathways to peripheral factors occurring within the muscle itself. However, it is assumed that subsequent fatigue after intensive exercise would account for about 80% of the impairments originating from a peripheral factor (Wiewelhove et al., 2017). Peripheral fatigue includes physical signs, such as the ultrastructural damage of connective tissue and muscle tissue, as well as an increase in muscle soreness. Nevertheless, previous research has indicated that ultrastructural damage does not always occur following intensive exercise that leads to muscle soreness (Yu et al., 2002). Therefore, it seems reasonable that reduced voluntary muscle activation (e.g., central fatigue due to inhibition caused by muscle soreness, swelling, and stiffness) also contributes to a reduction in muscular function (Byrne et al., 2004). Considering this, the recovery of dynamic performance measures with the use of post-rolling is due to either the facilitated process of soft-tissue restoration, the accelerated restoration of central factors, or both.

Although Pearcey et al. (2015) did not directly investigate the physiological mechanisms of FR, they speculated that post-rolling might enhance post-exercise recovery of dynamic performance measures via systemic biomechanical effects. These include: increased levels of circulating neutrophil; smaller increases in post-exercise plasma creatine kinase; activated mechano-sensory sensors that signal transcription of COX7B and ND1, indicating that new mitochondria are being formed, which presumably accelerate the healing of the muscle; and less active heat-shock proteins and immune cytokines, thus reflecting less cellular stress and inflammation. Furthermore, the reduced perception of pain may positively affect the short-term recovery process of muscular function (Zorko et al., 2016), and improved perception of muscle soreness may be critical for the restoration of exercise performance, since muscular function is impaired in the presence of muscle pain (Graven-Nielsen et al., 2002). These mechanisms will be described in more detail below.

However, the effects of post-rolling on performance should again be interpreted with caution, as the overall effects on sprint (*p* = 0.12) and strength performance (*p* = 0.28) were not significant and the number of available studies was limited. Merely one study found a clear benefit of post-rolling for sprint performance (D'Amico and Gillis, 2017), while only two studies showed post-rolling had a clear positive effect on strength performance (Zorko et al., 2016; Fleckenstein et al., 2017). In addition, the effects of post-rolling on jump capacity were negligible (Figure 7), although research has demonstrated a clear relationship between sprint, jump, and strength performance in athletes (Comfort et al., 2014). Therefore, it remains questionable whether the average post-rolling-induced enhancements of performance recovery were really due to a true physiological effect of FR or whether the placebo effect or methodological aspects contaminated these results.

The largest average effects of FR in general and post-rolling in particular were found for the alleviation of perceived muscle pain. The total Hedges' *g* of 0.47 (Figure 9) indicates that with the use of post-rolling, 66% of the population is likely to experience reduced muscle pain (Coe, 2002). In terms of athletic performance, muscle soreness, as previously described, can have negative consequences. It may result in altered muscle functions. These alterations may substantially reduce the performance or optimal training intensity of athletes (Pearcey et al., 2015). For example, Byrne et al. (2004) reported the negative effects of perceived muscle pain on sprint, jump, and strength performance, all of which are important during many athletic events.

Several theories have been proposed to explain the underlying mechanisms of exercise-induced muscle soreness. Some authors suggest that perceived muscle pain arises from disruption to the muscle fiber and surrounding connective tissue, while others suggest that it is associated with the inflammatory response, and other suggest that it is a combination of both (Hill et al., 2014). However, since muscle enzyme efflux and myofibrillar damage are not correlated with the actual sensation of muscle soreness, it has been postulated that exercise-induced muscle soreness may be more related to connective tissue damage and the inflammatory response rather than the actual muscle cell damage incurred (Macdonald et al., 2014). For example, damaged connective tissue stimulates mechanically sensitive receptors, giving rise to pain when stretched or pressed, while the inflammatory response, which follows tissue damage, creates an increase in tissue osmotic pressure that sensitizes the nociceptors, also resulting in the sensation of pain (Hill et al., 2014; Macdonald et al., 2014).

In this regard, FR-like treatments in animal models have been shown to induce an anti-nociceptive response by mediating an endogenous release of oxytocin into the plasma and in the central grey matter located around the cerebral aqueduct in the midbrain (Agren et al., 1995; Lund et al., 2002; Jay et al., 2014). Thus, one plausible mechanism to explain the reduction in muscle soreness following FR is the activation of descending inhibitory pathways, using the central gray matter-opioid system and oxytocin (Jay et al., 2014). Moreover, it has been proposed that FR-like mechanical stress may remove trigger points from the muscle tissue, leading to improved pain perception. Myofascial trigger points are a common source of musculoskeletal pain. It is thought that application of massage-like mechanical pressure on trigger points can prevent the unnecessary firing of muscle spindles afferent discharges from the trigger point, can reduce trigger point-induced muscle spasms, and ultimately decrease pain (Aboodarda et al., 2015). However, this explanation remains highly speculative because there is no concrete evidence for the effectiveness of brief rolling massages for trigger point therapy.

As proposed by Aboodarda et al. (2015) and Cavanaugh et al. (2017), the most plausible explanation for the mediation of perceived muscle pain following FR could be the effect of rolling massages on the central pain-modulatory systems. Their findings suggest that FR performed on muscles that contain a hypersensitive tender spot and FR performed on the contralateral muscle group can both provide an acute increase in the pain threshold. Since the increase in the pain threshold has a transient and non-localized effect, they suggest that massage-like mechanical pressure can provide analgesic effects through the ascending pain inhibitory system (gate theory of pain) and the descending anti-nociceptive pathway (diffuse noxious inhibitory control), respectively. Although the physiological mechanisms underlying the analgesic effect of FR have yet to be demonstrated empirically, a reduction in the sensation of muscle soreness is beneficial to athletes and may improve their readiness to participate in physical activity (Hill et al., 2014).

### Foam Rollers vs. Roller Massagers

Although pre-rolling effects were greater with the use of roller massagers, larger average percentage changes were seen with the use of foam rollers. Due to this contradictory finding, it is difficult to conclude whether pre-rolling with foam rollers or with roller massagers is superior. On the other hand, post-rolling showed both larger effects and greater percentage changes when it was administered with foam rollers, while the benefits of pre-rolling with roller massagers were less significant. Consequently, post-rolling seems to be more effective if foam rollers are used. However, as the number of high-quality and well-designed studies on FR is limited, the conclusions drawn above should be treated cautiously. Further studies would be necessary to confirm that different FR devices lead to different effects.

### Limitations

The results from the present meta-analysis provide evidence that the effects of FR on performance and recovery are rather minor and partly negligible, but can be relevant in some cases (e.g., to increase flexibility or to reduce muscle pain sensation). However, any meta-analysis is limited by the data available and there are several limitations for this analysis. First, most of the included studies contain small sample sizes, which result in reduced statistical power. Second, none of the included studies were able to blind their patients to the treatment due to the nature of the FR technique. As such, the placebo effect cannot be eliminated. Third, the methodology varied widely between the studies. For example, the majority of the published studies have not standardized and/or measured the applied pressure of the rolling action, while only a few studies have used a kind of pressure roller apparatus to maintain a constant rolling intensity. Consequently, there may be large ranges in the pressure exerted through FR. Furthermore, the FR procedures differed in terms of the duration of the application, the FR device used, and the muscles targeted. Fourth, with respect to the subjects' training status, the studies recruited both elite athletes and recreationally active or untrained individuals. Overall, these differences may explain some of the inconsistent findings within the current literature and future research should account for this inconsistency by at least directly measuring as well as reporting and standardizing the degree of pressure induced through FR. Finally, the statistical analysis is based only on articles that were published before January 2018. However, the vast majority of articles published since January 2018 confirm the pooled effects of FR, which are calculated in this meta-analysis. For example, Cheatham and Stull (2018), de Souza et al. (2019), Hall and Chadwick Smith (2018), Killen et al. (2018), Macgregor et al. (2018), Madoni et al. (2018), Monteiro et al. (2018, 2019a,b), and Smith et al. (2018, 2019) were able to confirm that FR can improve flexibility, perceived muscle pain perception, and/or the recovery of strength performance, while Madoni et al. (2018) found that FR had no effect on jump performance.

## Conclusion

In conclusion, this meta-analysis illustrates that pre-rolling seems to be an effective strategy for short-term improvements in flexibility without decreasing muscle performance. The review has also shown that the improvement of sprint performance to be expected from the use of pre-rolling, as well as the recovery rate of the performance measures of speed and strength with post-rolling, are significant enough to be relevant for at least elite athletes. The underlying mechanisms, however, remain elusive and the effects are in part contradictory. While the effects of FR on muscle function were less clear, the positive effects of alleviating muscle soreness with a larger body of evidence endorse the utilization of post-rolling. As psychological aspects play an important role in most sports, the fact that an athlete feels less pain after pre-rolling might be sufficient to justify its use despite the absence of measurable physiological benefits (Poppendieck et al., 2016). In addition, the almost complete absence of side effects might favor recovery-supporting FR intervention.

However, since the physiological mechanisms of the potential benefits of FR are not fully understood, care should be taken by athletes and coaches, particularly when considering that potential harmful side effects are also suggested when FR is applied (Freiwald et al., 2016). Further, it must be noted that in the available studies, different FR intervention protocols (e.g., different FR devices) were combined with different types of fatigue-inducing exercises and outcome measures, making it difficult to compare the results. In addition, there are not enough high-quality and well-designed studies on FR to draw any definite conclusions. Finally, due to the heterogeneity of the methodological designs among the included studies, there is no consensus on the optimal FR intervention (i.e., in terms of treatment time, pressure, and cadence, etc.). The existing literature thus provides some evidence to support the utilization of FR interventions in sports practice. However, the limited evidence should be considered prior to integrating foam rolling as a warm-up activity and/or a recovery tool.

## Author Contributions

TW and AD searched and reviewed studies, extracted and analyzed the data, and drafted and proofed the manuscript. CS contributed to data collection, statistical analyses, and reviewed the manuscript. LH reviewed and edited the manuscript. TM, MK, MP, and AF directed the project and contributed to discussion as well as reviewed and edited the manuscript.

### Conflict of Interest Statement

The authors declare that the research was conducted in the absence of any commercial or financial relationships that could be construed as a potential conflict of interest.
