# Supplementary material for: A Meta-Analysis of the Effects of Foam Rolling on Performance and Recovery
Source: Front Physiol. 2019 Apr 9;10:376. doi: 10.3389/fphys.2019.00376 (PMC6465761; doi:10.3389/fphys.2019.00376)
Supplement: Supplementary file 1 [file Table_1.pdf]

Table 1. Characteristics of the included studies on the effects of pre-rolling.

Author and year	Subjects (male/female)	FR intervention	Targeted muscles/region	Time between FR and post-test	Outcome measures	Effects
Sprint Performance						*Weighted overall:*<br>*+ 0.7% (g = 0.28)*
Healey et al., 2014	26 (13/13)<br>Healthy and recreationally active	1 x 30 s each muscle<br>Foam roller<br>Body weight	Quadriceps, hamstrings, iliotibial band, calves, latissimus dorsi, and rhomboid muscles	0 min	Pro-agility test	Post 0 min:<br>+ 0.2% (g = 0.02)
Mikesky et al., 2002	30 (7/23)<br>National Collegiate Athletic Asso-ciation Division II soccer, basket-ball, and volleyball athletes	A total of 2 min<br>Roller massager	Gluteals, hamstrings, quadriceps, and calf muscles	0 min	Flying-start 20-yard dash	Post 0 min:<br>+ 0.7 % (g = 0.66)
Peacock et al., 2014	11 (11/0)<br>Healthy, athletic, and physically active	1 x 30 s each region<br>Foam roller<br>Body weight	Thoracic/lumbar, gluteal, ham-string, calf, quadriceps/flexor, and pectoral regions	0 min	18.3-m pro agility test<br>37-m sprint test	Post 0 min:<br>+ 3.3% (g = 0.70)
Phillips et al., 2018	24 (8/16)<br>Healthy and physically active	1 x 5 min each muscle<br>Foam roller<br>Body weight	Quadriceps and triceps surae mus-cle groups	0 min	Pro-agility test	Post 0 min:<br>- 0.7% (g = -0.09)
Jump Performance						*Weighted overall:*<br>*- 1.9% (g = 0.09)*
Grabow et al., (2017)	16 (8/8)<br>Healthy and either resistance and/or aerobically physically trained	3 x 60 s<br>Roller massager<br>90 % of the participants maximum rating of perceived pain	Quadriceps muscles	0 min<br><br>10 min	Vertical single-leg drop jump test	Post 0 min:<br>- 3.0% (g = -0.02)<br>Post 10 min:<br>+ 1.8% (g = 0.01)
Healey et al., 2014	26 (13/13)<br>Healthy and recreationally active	1 x 30 s each muscle<br>Foam roller<br>Body weight	Quadriceps, hamstrings, iliotibial band, calves, latissimus dorsi, and rhomboid muscles	0 min	Vertical counter-movement jump test	Post 0 min:<br>+ 2.5% (g = 0.16)
Jones et al., (2015)	20 (20/0)<br>Healthy and recreationally active	1 x 30 s each muscle<br>Foam roller<br>Body weight	Gastrocnemii, quadriceps, ham-strings, and glutei muscles	0 min	Vertical jump test	Post 0 min:<br>- 0.3% (g = -0.02)

Table 1. Continued.

Author and year	Subjects (male/female)	FR intervention	Targeted muscles/region	Time between FR and post-test	Outcome measures	Effects
Mikesky et al., 2002	30 (7/23)<br>National Collegiate Athletic Association Division II soccer, basketball, and volleyball athletes	A total of 2 min<br>Roller massager	Gluteals, hamstrings, quadriceps, and calf muscles	0 min	Vertical counter-movement jump test	Post 0 min:<br>+ 1.0% (g = 0.36)
Peacock et al., 2014	11 (11/0)<br>Healthy, athletic, and physically active	1 x 30 s each region<br>Foam roller<br>Body weight	Thoracic/lumbar, gluteal, hamstring, calf, quadriceps/flexor, and pectoral regions	0 min	Vertical counter-movement jump test<br>Standing long jump test	Post 0 min:<br>+ 4.9% (g = 0.43)
Phillips et al., 2018	24 (8/16)<br>Healthy and physically active	1 x 5 min each muscle<br>Foam roller<br>Body weight	Quadriceps and triceps surae muscle groups	0 min	Vertical counter-movement jump test	Post 0 min:<br>- 2.6% (g = -0.13)
Sagiroglu et al., 2017	16 (16/0)<br>Well-trained combat athletes, who were competing in Judo, Karate, Tae Kwon Do, and Muay Thai	2 x 30 s each muscle<br>Foam roller<br>Body weight	Hamstrings, quadriceps, hip, and gastrocnemius muscles	0 min<br>5 min<br>10 min<br>15 min<br>30 min	Vertical counter-movement jump test	Post 0 min:<br>- 4.2% (g = -0.50)<br>Post 5 min<br>- 3.7% (g = -0.44)<br>Post 10 min:<br>- 0.2 % (g = -0.03)<br>Post 15 min:<br>- 0.5% (g = -0.06)<br>Post 30 min:<br>- 1.1% (g = -0.14)
Strength Performance						*Weighted Overall:*<br>*+ 1.8% (g = 0.12)*
Cavanaugh et al., 2017	12 (12/0)<br>Healthy and recreationally active	3 x 30 s<br>Roller massager<br>7 on a visual analog pain scale of 1 to 10	Gastrocnemius muscle	0 min<br>5 min	Maximum isometric voluntary force of the plantar flexors	Post 0 min:<br>- 0.8% (g = -0.04)<br>Post 5 min:<br>- 3.6% (g = -0.19)
Grabow et al., 2017	16 (8/8)<br>Healthy and either resistance and/or aerobically physically trained	3 x 60 s<br>Roller massager<br>90% of the participants maximum rating of perceived pain	Quadriceps muscles	0 min<br>10 min	Maximum isometric voluntary force of the knee extensors and knee flexors	Post 0 min:<br>- 4.7% (g = -0.04)<br>Post 10 min:<br>+ 2.8% (g = 0.04)

Table 1. Continued.

Author and year	Subjects (male/female)	FR intervention	Targeted muscles/region	Time between FR and post-test	Outcome measures	Effects
Healey et al., 2014	26 (13/13)<br>Healthy and recreationally active	1 x 30 s each muscle<br>Foam roller<br>Body weight	Quadriceps, hamstrings, iliotibial band, calves, latissimus dorsi, and rhomboid muscles	0 min	Maximum isometric voluntary quarter squat force	Post 0 min:<br>+ 3.9% (g = 0.13)
MacDonald et al., 2013	11 (11/0)<br>Healthy, recreationally active, and resistance trained	2 x 1 min<br>Foam roller<br>Body weight	Quadriceps muscles	0 min<br><br>10 min	Maximum isometric voluntary force of the knee extensors	Post 0 min:<br>+ 11.5% (g = 0.87)<br>Post 10 min:<br>+ 7.0% (g = 0.55)
Mikesky et al., 2002	30 (7/23)<br>National Collegiate Athletic Association Division II soccer, basketball, and volleyball athletes	A total of 2 min<br>Roller massager	Gluteals, hamstrings, quadriceps, and calf muscles	0 min	Maximum isokinetic voluntary concentric force of the knee extensors	Post 0 min:<br>+ 0.3% (g = 0.11)
Peacock et al., 2014	11 (11/0)<br>Healthy, athletic, and physically active	1 x 30 s each region<br>Foam roller<br>Body weight	Thoracic/lumbar, gluteal, hamstring, calf, quadriceps/flexor, and pectoral regions	0 min	Indirect 1RM bench press	Post 0 min:<br>+ 3.8% (g = 0.18)
Sullivan et al., 2013	17 (7/10)<br>Healthy and recreationally active	2 x 10 s<br>Roller massager<br>13 kg using a constant pressure roller apparatus	Hamstring muscles	0 min	Maximum isometric voluntary force of the knee flexors	Post 0 min:<br>0.0% (g = 0.00)
Overall Performance						*Weighted overall:*<br>*+ 1.5% (g = 0.20)*
Flexibility						*Weighted overall:*<br>*+ 4.0% (g = 0.34)*
Bushell et al., 2015	31 (19/12)<br>Healthy and competitive or recreational athletes	3 x 1 min<br>Foam roller<br>Body weight	Quadriceps muscles	0 min	Hip joint range of motion	Post 0 min:<br>+ 1.2% (g = 0.33)
Cheatham et al., 2017	45 (27/18)<br>Healthy and recreationally active	1 x 2 min<br>Foam roller	Quadriceps muscles	0 min	Knee joint range of motion	Post 0 min:<br>+ 2.9% (g = 0.37)

Table 1. Continued.

Author and year	Subjects (male/female)	FR intervention	Targeted muscles/region	Time between FR and post-test	Outcome measures	Effects
Grabow et al., 2017	16 (8/8)<br>Healthy and either resistance and/or aerobically physically trained	3 x 60 s<br>Roller massager<br>90 % of the participants maximum rating of perceived pain	Quadriceps muscles	0 min<br><br>10 min	Knee joint range of motion	Post 0 min:<br>+ 1.9% (g = 0.15)<br>Post 10 min:<br>+ 2.9% (g = 0.22)
Griefahn et al., 2017	38 (25/13)<br>Healthy and physically active	3 x 30 s each muscle<br>Foam roller<br>Body weight	Gluteus maximus, erector spinae, and latissimus dorsi muscles	0 min	Modified schober test	Post 0 min:<br>+ 0.2% (g = 0.01)
MacDonald et al., 2013	11 (11/0)<br>Healthy, recreationally active, and resistance trained	2 x 1 min<br>Foam roller<br>Body weight	Quadriceps muscle	0 min<br><br>10 min	Knee joint range of motion	Post 0 min:<br>+ 11.5% (g = 0.87)<br>Post 10 min:<br>+ 7.0% (g = 0.55)
Mikesky et al., 2002	30 (7/23)<br>National Collegiate Athletic Association Division II soccer, basketball, and volleyball athletes	A total of 2 min<br>Roller massager	Gluteals, hamstrings, quadriceps, and calf muscles	0 min	Hamstring flexibility	Post 0 min:<br>+ 1.1% (g = 0.49=
Murray et al., 2016	12 (12/0)<br>Squash players from an elite	1 x 60 s<br>Foam roller<br>Body weight	Quadriceps muscle	0 min<br><br>5 min<br><br>10 min<br><br>15 min<br><br>30 min	Hip flexor and quadriceps flexibility	Post 0 min:<br>+ 0.4% (g = 0.05)<br>Post 5 min:<br>+ 1.4% (g = 0.14)<br>Post 10 min:<br>+ 1.4% (g = 0.13)<br>Post 15 min:<br>+ 1.8% (g = 0.22)<br>Post 30 min:<br>- 1.1 % (g = -0.13)
Peacock et al., 2014	11 (11/0)<br>Healthy, athletic, and physically active	1 x 30 s each region<br>Foam roller<br>Body weight	Thoracic/lumbar, gluteal, hamstring, calf, quadriceps/flexor, and pectoral regions	0 min	Sit-and-reach test	Post 0 min:<br>+ 0.4% (g = 0.02)
Phillips et al., 2018	24 (8/16)<br>Healthy and physically active	1 x 5 min each muscle<br>Foam roller<br>Body weight	Quadriceps and triceps surae muscle groups	0 min	Knee joint range of motion	Post 0 min:<br>+ 19.2% (g = 0.82)

Table 1. Continued.

Author and year	Subjects (male/female)	FR intervention	Targeted muscles/region	Time between FR and post-test	Outcome measures	Effects
Sagiroglu et al., 2017	16 (16/0)<br>Well-trained combat athletes, who were competing in Judo, Karate, Tae Kwon Do, and Muay Thai	2 x 30 s each muscle<br>Foam roller<br>Body weight	Hamstrings, quadriceps, hip, and gastrocnemius muscles	0 min<br><br>5 min<br><br>10 min<br><br>15 min<br><br>30 min	Sit-and-reach test	Post 0 min:<br>+ 5.8% (g = 0.10)<br>Post 5 min:<br>+ 3.6% (g = 0.07)<br>Post 10 min:<br>+ 0.2% (g = 0.02)<br>Post 15 min:<br>- 3.7% (g = -0.04)<br>Post 30 min:<br>- 11.5% (g = -0.17)

FR, foam rolling; 1RM, one repetition maximum.
