# Supplementary material for: A Meta-Analysis of the Effects of Foam Rolling on Performance and Recovery
Source: Front Physiol. 2019 Apr 9;10:376. doi: 10.3389/fphys.2019.00376 (PMC6465761; doi:10.3389/fphys.2019.00376)
Supplement: Supplementary file 2 [file Table_2.pdf]

Table 2. Characteristics of the included studies on the effects of post-rolling.

**Author and year**	**Subjects (male/female)**	**Exercise to provoke exhaustion**	**FR intervention**	**Targeted muscles/region**	**Time between FR and post-test**	**Outcome measures**	**Effects**
Sprint Performance							*Weighted Overall:*<br>*+ 3.1% (g = 0.34)*
D’Amico and Gillis, 2017	37 (37/0)<br>Healthy	40 x 15 m sprints with a 5 m deceleration zone	2 x 60 s each muscle<br>Foam roller<br>Body weight	Quadriceps, hamstrings, gluteus maximus, and gastrocnemius muscles	0 h<br>24 h<br>48 h<br>72 h<br>96 h	T-test	Post 0 h:<br>+ 4.0% (g = 0.35)<br>Post 24 h:<br>+ 6.2% (g = 0.55)<br>Post 48 h:<br>+ 6.9% (g = 0.61)<br>Post 72 h:<br>+ 3.3% (g = 0.29)<br>Post 96 h:<br>+ 4.5% (g = 0.41)
Pearcey et al., 2015	8 (8/0)<br>Healthy and moderately to very physically active	10 sets of 10 repetitions of barbell back squats at 60% of the participant`s 1RM	2 x 45 s each Muscle<br>Foam roller<br>Body weight	Quadriceps, adductors, hamstrings, iliotibial band, and gluteus muscles	24 h<br>48 h<br>72 h	30-m sprint test<br>T-test	Post 24 h:<br>- 0.4% (g = 0.09)<br>Post 48 h:<br>+ 0.2% (g = 0.00)<br>Post 72 h:<br>+ 0.9% (g = 0.27)
Rey et al., 2017	18 (18/0)<br>Spanish professional soccer players	Standard soccer training	2 x 45 s each Muscle<br>Foam roller<br>Body weight	Quadriceps, hamstrings, adductors, gluteals, and gastrocnemius muscles	24 h	10-m sprint test<br>T-test	Post 24 h:<br>+ 2.2% (g = 0.37)
Jump Performance							*Weighted Overall:*<br>*- 0.2% (g = 0.06)*
D’Amico and Gillis, 2017	37 (37/0)<br>Healthy	40 x 15 m sprints with a 5 m deceleration zone	2 x 60 s each muscle<br>Foam roller<br>Body weight	Quadriceps, hamstrings, gluteus maximus, and gastrocnemius muscles	0 h<br>24 h<br>48 h<br>72 h<br>96 h	Vertical squat jump test	Post 0 h:<br>- 1.0% (g = 0.02)<br>Post 24 h:<br>+ 1.3% (g = 0.17)<br>Post 48 h:<br>+ 1.0% (g = 0.14)<br>Post 72 h:<br>- 1.9% (g = -0.04)<br>Post 96 h:<br>- 0.8% (g = 0.02)

Table 2. Continued.

**Author and year**	**Subjects (male/female)**	**Exercise to provoke exhaustion**	**FR intervention**	**Targeted muscles/region**	**Time between FR and post-test**	**Outcome measures**	**Effects**
Fleckenstein et al., 2017	30 (15/15)<br>Healthy and physically active	Functional agility short-term fatigue protocol (FAST-FP)	1 x 30 s each muscle<br>Foam roller<br>7 on a visual analog pain scale of 1 to 10	Quadriceps, hamstrings, adductors, calves, and iliotibial band muscles	5 min	Reactive strength index in a drop jump test	Post 5 min:<br>- 7.1% (g = -0.28)
Macdonald et al., 2014	20 (20/0)<br>Physically active and resistance trained	10 sets of 10 repetitions of barbell back squats at 60% of the participant`s 1RM	2 x 60 s each muscle<br>Foam roller<br>Body weight	Muscle groups of the anterior, lateral, posterior, and medial aspect of the thigh, along with the gluteal muscles	24 h<br>48 h<br>72 h	Vertical counter-movement jump test	Post 24 h:<br>+ 6.0% (g = 0.36)<br>Post 48 h:<br>+ 5.9% (g = 0.36)<br>Post 72 h:<br>0.0% (g = 0.00)
Pearcey et al., 2015	8 (8/0)<br>Healthy and moderately to very physically active	10 sets of 10 repetitions of barbell back squats at 60% of the participant`s 1RM	2 x 45 s each Muscle<br>Foam roller<br>Body weight	Quadriceps, adductors, hamstrings, iliotibial band, and gluteus muscles	24 h<br>48 h<br>72 h	Standing broad jump test	Post 24 h:<br>+ 2.6% (g = 0.22)<br>Post 48 h:<br>+ 4.7% (g = 0.38)<br>Post 72 h:<br>+ 5.4% (g = 0.44)
Rey et al., 2017	18 (18/0)<br>Spanish professional soccer players	Standard soccer training	2 x 45 s each Muscle<br>Foam roller<br>Body weight	Quadriceps, hamstrings, adductors, gluteals, and gastrocnemius muscles	24 h	Vertical counter-movement jump test	Post 24 h:<br>+ 2.7% (g = 0.18)
Strength Performance							*Weighted Overall:*<br>*+ 3. % (g = 0.21)*
Casanova et al., 2018	10 (8/2)<br>Healthy athletes with occasional experience of resistance training	5 sets of 20 repetitions of unilateral calf raises within the maximal active range of motion	6 x 45 s<br>Roller massager	Gastrocnemius muscle	1 h<br>24 h<br>48 h<br>72 h	Maximum isometric voluntary force of the plantar flexors	Post 1 h:<br>+ 6.6% (g = 0.18)<br>Post 24 h:<br>- 1.2% (g = -0.03)<br>Post 48 h:<br>- 3.1% (g = -0.07)<br>Post 72 h:<br>- 5.4% (g = -0.13)

Table 2. Continued.

Author and year	Subjects (male/female)	Exercise to provoke exhaustion	FR intervention	Targeted muscles/region	Time between FR and post-test	Outcome measures	Effects
Fleckenstein et al., 2017	30 (15/15)<br>Healthy and physically active	Functional agility short-term fatigue protocol (FAST-FP)	1 x 30 s each muscle<br>Foam roller<br>7 on a visual analog pain scale of 1 to 10	Quadriceps, hamstrings, adductors, calves, and iliotibial band muscles	5 min	Maximum isometric voluntary force of the knee extensors	Post 5 min:<br>+ 9.2% (g = 0.61)
Macdonald et al., 2014	20 (20/0)<br>Physically active resistance-trained	10 sets of 10 repetitions of barbell back squats at 60 % of the participant`s 1RM	2 x 60 s each muscle<br>Foam roller<br>Body weight	Muscle groups of the anterior, lateral, posterior, and medial aspect of the thigh, along with the gluteal muscles	24 h<br>48 h<br>72 h	Maximum isometric voluntary force of the knee extensors	Post 24 h:<br>+ 3.0% (g = 0.05)<br>Post 48 h:<br>0.0% (g = -0.07)<br>Post 72 h:<br>- 1.0% (g = -0.13)
Pearcey et al., 2015	8 (8/0)<br>Healthy and moderately to very physically active	10 sets of 10 repetitions of barbell back squats at 60 % of the participant`s 1RM	2 x 45 s each Muscle<br>Foam roller<br>Body weight	Quadriceps, adductors, hamstrings, iliotibial band, and gluteus muscles	24 h<br>48 h<br>72 h	Barbell back squats at 70 % of the participant`s 1RM for a maximal number of repetitions	Post 24 h:<br>+ 1. % (g = 0.04)<br>Post 48 h:<br>+ 14.1% (g = 0.37)<br>Post 72 h:<br>+ 3.7% (g = 0.10)
Zorko et al., 2016	10 (9/1)<br>Recreationally active	3 sets of 15 repetitions of knee extensions at 70 % of the participant`s 1 RM	1 x 90 s<br>Foam roller<br>Body weight	Quadriceps muscle	30 min	Maximum isometric voluntary force of the knee extensors	Post 30 min:<br>+ 4.9% (g = 0.24)
Overall Performance							*Weighted Overall:*<br>*+ 1.9% (g = 0.19)*
Muscle pain							*Weighted Overall:*<br>*+ 6.0% (g = 0.47)*
Casanova et al., 2018	10 (8/2)<br>Healthy athletes with occasional experience of resistance training	5 sets of 20 repetitions of unilateral calf raises within the maximal active range of motion	6 x 45 s<br>Roller massager	Gastrocnemius muscle	1 h<br>24 h<br>48 h<br>72 h	Pressure-pain threshold	Post 1 h:<br>+ 7.2% (g = 0.21)<br>Post 24 h:<br>+ 0.2% (g = 0.04)<br>Post 48 h:<br>+ 6.1% (g = 0.21)<br>Post 72 h:<br>+ 9.7% (g = 0.32)

Table 2. Continued.

**Author and year**	**Subjects (male/female)**	**Exercise to provoke exhaustion**	**FR intervention**	**Targeted muscles/region**	**Time between FR and post-test**	**Outcome measures**	**Effects**
D'Amico and Gillis, 2017	37 (37/0)<br>Healthy	40 x 15 m sprints with a 5 m deceleration zone	2 x 60 s each muscle<br>Foam roller<br>Body weight	Quadriceps, hamstrings, gluteus maximus, and gastrocnemius muscles	0 h<br><br>24 h<br><br>48 h<br><br>72 h<br><br>96 h	General labeled magnitude scale (gLMS)	Post 0 h:<br>- 4.8% (g = -0.50)<br>Post 24 h:<br>- 1.4% (g = -0.15)<br>Post 48 h:<br>+ 1.8% (g = 0.19)<br>Post 72 h:<br>+ 1.2% (g = 0.12)<br>Post 96 h:<br>- 3.0% (g =-0.31)
Macdonald et al., 2014	20 (20/0)<br>Physically active resistance-trained	10 sets of 10 repetitions of barbell back squats at 60 % of the participant`s 1RM	2 x 60 s each muscle<br>Foam roller<br>Body weight	Muscle groups of the anterior, lateral, posterior, and medial aspect of the thigh, along with the gluteal muscles	24 h<br><br>48 h<br><br>72 h	BS-11 numerical rating scale	Post 24 h:<br>+ 11.0% (g = 1.00)<br>Post 48 h:<br>+ 27.0% (g = 2.46)<br>Post 72 h:<br>+ 25.0% (g = 2.28)
Pearcey et al., 2015	8 (8/0)<br>Healthy and moderately to very physically active	10 sets of 10 repetitions of barbell back squats at 60 % of the participant`s 1RM	2 x 45 s each Muscle<br>Foam roller<br>Body weight	Quadriceps, adductors, hamstrings, iliotibial band, and gluteus muscles	24 h<br><br>48 h<br><br>72 h	Pressure-pain threshold	Post 24 h:<br>+ 7.6% (g = 0.28)<br>Post 48 h:<br>+ 11.0% (g = 0.42)<br>Post 72 h:<br>+ 0.7% (g = 0.02)
Rey et al., 2017	18 (18/0)<br>Spanish professional soccer players	Standard soccer training	2 x 45 s each Muscle<br>Foam roller<br>Body weight	Quadriceps, hamstrings, adductors, gluteals, and gastrocnemius muscles	24 h	Visual analog scale	Post 24 h:<br>+ 15.3% (g = 1.98)

FR, foam rolling; 1RM, one repetition maximum.
